# A Light-Powered Liquid Crystal Elastomer Roller

**DOI:** 10.3390/polym15214221

**Published:** 2023-10-25

**Authors:** Kai Li, Jiajing Chen, Haoyu Hu, Haiyang Wu, Yuntong Dai, Yong Yu

**Affiliations:** School of Civil Engineering, Anhui Jianzhu University, Hefei 230601, China; kli@ahjzu.edu.cn (K.L.); jiajingchen2022@126.com (J.C.); haoyuhu2022@163.com (H.H.); haiyangwu2022@163.com (H.W.); daiytmechanics@ahjzu.edu.cn (Y.D.)

**Keywords:** roller, self-rolling, liquid crystal elastomer, light-powered, fiber

## Abstract

Achieving and controlling the desired movements of active machines is generally accomplished through precise control of artificial muscles in a distributed and serialized manner, which is a significant challenge. The emerging motion control strategy based on self-oscillation in active machines has unique advantages, including directly harvesting energy from constant ambient light, and it has no need for complex controllers. Inspired by the roller, we have innovatively developed a self-rolling roller that consists of a roller and a liquid crystal elastomer (LCE) fiber. By utilizing a well-established dynamic LCE model and subjecting it to constant illumination, we have investigated the dynamic behavior of the self-rolling roller. Based on numerical calculations, it has been discovered that the roller, when subjected to steady illumination, exhibits two distinct motion regimes: the static regime and the self-rolling regime. The self-rolling regime, characterized by continuous periodic rolling, is sustained by the interaction between light energy and damping dissipation. The continuous periodic rolling observed in the self-rolling regime is maintained through the interplay between the dissipation of damping and the absorption of light energy. In the static state, the rolling angle of the roller begins to decrease rapidly and then converges to zero. Detailed investigations have been conducted to determine the critical conditions required to initiate self-rolling, as well as the essential system parameters that influence its frequency and amplitude. The proposed self-rolling roller has superiorities in its simple structure, light weight, alternative to manual labor, and speediness. This advancement is expected to inspire greater design diversity in micromachines, soft robotics, energy harvesters, and similar areas.

## 1. Introduction

Active materials are materials with special physical or chemical properties that can undergo reversible or irreversible physical or chemical changes under external stimuli, and active materials are capable of responding to various external stimuli by undergoing deformation and movement [1]. Active materials can be utilized as artificial muscles to drive active machines [2], which offers several unique advantages over traditionally motor-driven machines, including integration of the driving unit and structural unit for lighter weight, noiseless operation during motion work through deformation, low modulus and environmental friendliness, and impact resistance and recoverability [3,4,5]. An active machine can be defined as a machine that is running, producing energy, or performing a task. These machines, which often require an external energy supply, which can be controlled and manipulated using electrical, magnetic, and light stimuli, provide several advantages, including wireless remote control and the ability to navigate through barriers [6,7,8]. Active machines have shown tremendous potential in a variety of fields, including soft robotics, medical devices, and micro-nano devices, due to the numerous benefits they provide. These benefits make them highly promising and sought after in these areas [9,10,11].

Currently, achieving and controlling the desired movements of active machines is a significant challenge. The conventional solution is to achieve this through precise control of artificial muscles in a distributed and serialized manner, similar to how the brain controls the distributed muscle system in organisms [2,3,4,5]. With the rapid development of artificial intelligence, it is expected that the fusion of machine learning and artificial muscles will enable more intelligent artificial mobile robots [7]. This motion control strategy requires timely perception of motion and environmental states, as well as precise control of the deformation of artificial muscles, which is similar to how the brain controls the distributed muscles in the bodies of animals, adjusting them in real-time to achieve motion. Therefore, it requires a central nervous system capable of complex control. The complexity and requirements for control accuracy pose significant challenges that greatly limit the application range of active machines.

Although this complex control method is necessary for accomplishing complex motion tasks, in engineering applications, periodic motion patterns are often used. The motion control strategy based on self-oscillation in active machines has unique advantages in achieving common periodic motion patterns [12,13,14,15,16,17,18]. Self-oscillation refers to the phenomenon of periodic oscillation that is initiated by a steady external stimulus [19]. By continuously and periodically extracting energy from a constant external source, self-oscillation can be sustained in a continuous and periodic manner. This feature, to some extent, simplifies the complexity of self-oscillating systems and enables fascinating applications, including portability. One significant advantage of self-oscillation is that the system parameters have a greater influence on determining the period and amplitude of the oscillation compared to the initial conditions. This property enhances the robustness of self-oscillating systems [20,21]. These superiorities of self-oscillating machines are driving them to become attractive candidates for a variety of practical applications, including active machines [22,23,24,25,26,27,28], autonomous robotics [29], energy-absorbing devices [30,31], motors [32], etc.

Diverse self-oscillating systems have been successfully developed by utilizing a range of responsive materials, including liquid crystal elastomers (LCEs) [33], ionic gels [34,35], hydrogels [36,37], and others. Moreover, many attempts have been made to construct several self-sustained motion modes that have been observed in self-oscillating systems, including vibration [38,39,40], bending [41,42], rolling [5,43,44], spinning [45], torsion [46,47], stretching [48], self-oscillation auxetic metamaterials [49,50], self-floating [51], eversion or inversion [52], curling [53], swimming [54], buckling [55,56,57], jumping [58,59], rotation [60], chaos [61], and the self-sustained collective motion of a spring oscillator [62]. Additionally, the synchronized motion of multiple coupled self-oscillators has also been achieved in certain systems [63,64,65].

In self-oscillating systems, it is essential to incorporate specific mechanisms that can absorb energy from the external environment. This is necessary to compensate for the energy consumed by system damping [18]. In combination with different responsive materials, these mechanisms include coupling mechanisms between chemical reactions and large deformations [34,35], self-shading mechanisms [22,23,66], and the coupling mechanisms between droplet evaporation and movement involve multiple processes [66]. LCE is a crucial type of optically responsive material that is created by combining anisotropic rod-like liquid crystal molecules with stretchable long-chain polymers [67]. This material exhibits significant changes in its configuration and macroscopic deformation in response to environmental stimuli such as light, heat, electricity, and magnetism. These changes occur due to rotations or phase transitions of the liquid crystal monomer molecules within the LCE structure. Optically responsive LCE exhibits a rapid response, significant intrinsic deformation, and the ability to undergo reversible deformation [68].

**Figure 1:**
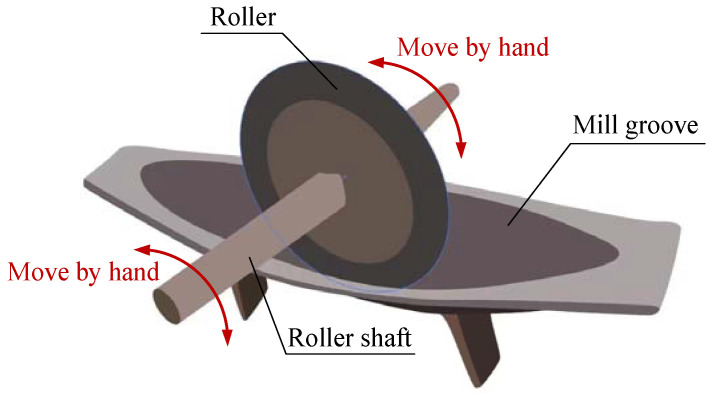
Schematic diagram of a roller. Rolling of the roller is achieved by rhythmically controlling the movement of the roller shaft.

Although the tremendous interest in LCE-based self-oscillating systems has led to the construction of many self-oscillating systems, there is still a need to build self-oscillating machines with more diverse motion patterns in order to meet the increasing demands of various functional applications. The roller is a tool that has been circulated for a relatively long time, which is mainly composed of roller, roller shaft, and mill groove. It can rhythmically control the translational motion of the roller shaft and drive the rolling of the roller, as shown in Figure 1. This paper presents a novel roller design utilizing LCE fiber and demonstrates its self-oscillating vibration under steady illumination. The roller’s unique advantages, including its simple structure, ability to replace manual labor, speediness, and lightweight nature, make it a promising tool for expanding design possibilities in a wide range of fields, including soft robotics, energy harvesting, and micromachines.

The remaining sections of this paper are organized as follows. In Section 2, we introduce a nonlinear dynamic model for the self-rolling roller under steady illumination. This model incorporates the well-established dynamic LCE model and provides the governing equations for the system. Moving on to Section 3, we explore two distinct motion regimes observed in the self-rolling roller under steady illumination and delve into the underlying mechanism of self-rolling. Section 4 is dedicated to investigating the critical conditions that initiate self-rolling and analyzing the effects of different system parameters on the frequency and amplitude of the roller’s motion. Finally, in Section 5, we summarize our findings and conclude the study.

## 2. Model and Formulation

In this section, we develop a dynamic model for the self-rolling LCE roller under steady illumination by combining the rigid-body pure rolling model and the LCE dynamic model. This model encompasses several essential components, including the dynamic oscillations of the self-rolling roller, the tension experienced by the LCE fiber, the changes in the number fraction of *cis*-isomers within the LCE fiber, the process of nondimensionalization, and the solution technique for the differential equations governing the system, which have variable coefficients.

### 2.1. Dynamics of an LCE Roller

**Figure 2:**
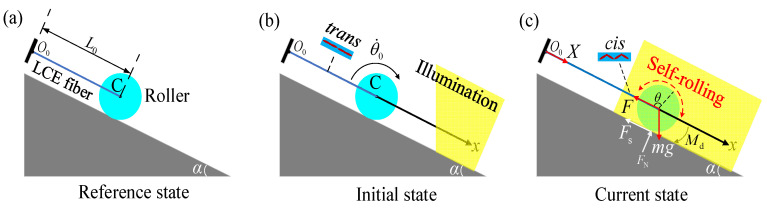
Schematics of a self-rolling roller: (**a**) reference state, (**b**) initial state, and (**c**) current state. Under steady illumination, the LCE fiber undergoes light-driven contraction while in motion, resulting in self-rolling of the roller due to the interplay between the fiber’s motion and contraction.

In this section, we develop the dynamic model and establish the governing equations for the self-rolling LCE roller under steady illumination. A roller is a traditional manual device with wheel rotation around the vertical axis and its own central axis; there is a pure rolling section, the inner side of which slides backward, and the outer forward to crush grain. Due to the low speed of roller rotation, the influence of the gyroscopic moment is less. Based on this inspiration, this article develops a self-rolling system, which contains a roller of mass *m* and radius *R* and an LCE fiber under steady illumination. As illustrated in Figure 2a, one end of the LCE fiber is fixed at point $O_{0}$, while the other end is linked to the center C of the roller.

In the reference state, the original length of the LCE fiber is $L_{0}$ and there is no stress applied to it. The photosensitive molecules such as azobenzene molecules in the nematic LCE fiber are oriented along the fiber axis. It is a well-known phenomenon that the LCE fibers will contract under illumination due to the transformation of azobenzene liquid crystal molecules from a straight *trans* state to a bent *cis* state, while the light-driven contraction will recover in darkness due to the transformation of azobenzene liquid crystal molecules from a bent *cis* state to a straight *trans* state [31,69]. Figure 2b illustrates the initial state of the roller. The initial velocity of the roller is denoted by ${\dot{\theta }}_{0}$. As depicted in Figure 2c, in the current state, only the right half of the center C of the roller is steadily illuminated, while the rest of the roller is in darkness. As the roller rolls down, the connecting segments of the LCE fiber contract under illumination due to the transformation of azobenzene liquid crystal molecules from a straight *trans* state to a bent *cis* state. This contraction in the LCE fiber, driven by light, results in an increased tension F(t) within the fiber. The friction $F_{s}$ between the roller and the inclined plane generates a rolling moment M(t) that consistently opposes the rotation of the roller in the opposite direction. As the rolling angle of the roller increases, the connection segment exposed to illumination gradually increases, and the tension F(t) also increases significantly with the increase in the connection segment exposed to illumination. Subsequently, the roller speed decreases and the roller is driven to rotate above the inclined plane. As the roller rolls in the opposite direction, the LCE fiber gradually exits the illuminated area, and the contraction of the LCE fiber recovers due to that the azobenzene liquid crystal molecules within the LCE fiber revert from bent *cis* state to straight *trans* state. Due to its own inertia, the roller continues to roll past the equilibrium position. As a result, the LCE fiber returns to the dark area in the opposite direction. Finally, under steady illumination, the roller may exhibit continuous periodic motion.

The roller experiences both a rolling moment M(t) and a damping moment $M_{d}\left(t\right)$. To simplify the analysis, we make the assumption that the damping moment $M_{d}\left(t\right)$ of the roller is directly proportional to its rolling angular velocity $\dot{\theta }$. Consequently, we can derive the nonlinear dynamic governing equation of the self-rolling roller as follows:

(1) $$J\ddot{\theta }=M\left(t\right)-M_{f}\mathrm{sgn}\left(\dot{\theta }\right)-\beta_{1}\dot{\theta }-\beta_{2}V_{c},$$

(2) $$ma_{c}=mg\sin\alpha -F\left(t\right)-F_{s},$$

where g represents the gravitational acceleration, $\beta_{1}$ represents the rotational damping coefficient, $\beta_{2}$ represents the translational damping coefficient, and $V_{c}$ represents the translational velocity of the roller, calculated as $V_{c}=R\dot{\theta }$, and $a_{c}$ is the translational acceleration of the roller, calculated as $a_{c}=R\ddot{\theta }$. *J* represents the moment of inertia of the roller around its center point *C*, calculated as $J=1/2mR^{2}$, $\dot{\theta }$ and $\ddot{\theta }$ denote the angular velocity and rolling acceleration of the roller.

It should be emphasized that the rolling moment M(t) mentioned in Equation (1) arises from the friction $F_{s}$ occurring between the roller and the inclined plane. This frictional force can be calculated using the following formula:

(3) $$M\left(t\right)=F_{s}R=mgR\sin\alpha -F\left(t\right)R-mR^{2}\ddot{\theta }.$$

Substituting Equation (3) into Equation (1) and ignoring the roll resistance, we have

(4) $$3/2mR^{2}\ddot{\theta }=mgR\sin\alpha -F\left(t\right)R-\beta \dot{\theta },$$

where β is the damping coefficient, which is the combination of the rotation damping coefficient and the translational damping coefficient.

### 2.2. Tension of LCE Fibers

To calculate the rolling moment M(t) mentioned in Equations (1) and (3), it is necessary to determine the tension of the LCE fiber F(t). The tension F(t) is uniformly distributed throughout the entire length of the LCE fiber. For simplicity, the mass of the LCE fiber is considered to be negligible, and the cross-sectional shape of the LCE fiber is assumed to remain constant when subjected to tension. To analyze the non-uniform deformations of the LCE fibers, we utilize two coordinate systems: the Lagrangian arc coordinate system *X*, which represents the reference state (Figure 2a), and the Eulerian arc coordinate system *x*, which describes the current state (Figure 2c). During the oscillation of the roller, we can express the position of a material point within the LCE fiber as *x* = *x*(*X*, *t*), where *X* represents the Lagrangian arc coordinate and t represents time. The thickness δ of the LCE fiber is assumed to be much smaller than the penetration depth $d_{0}$ of light, and thus light-driven contraction $\varepsilon_{L}$ is denoted as $\varepsilon_{L}\left(X,t\right)$. The total strain $\varepsilon_{\mathrm{tot}}$ in the LCE fiber can be represented as $\varepsilon_{\mathrm{tot}}\left(X,t\right)$. For small deformations, the total strain $\varepsilon_{\mathrm{tot}}\left(X,t\right)$ is the sum of the light-driven contraction $\varepsilon_{L}\left(X,t\right)$ and the elastic strain, i.e.,

(5) $$\varepsilon_{e}\left(t\right)=\varepsilon_{\mathrm{tot}}\left(X,t\right)+\varepsilon_{L}\left(X,t\right).$$

It should be noted that $\varepsilon_{e}\left(t\right)$ is homogeneous throughout the LCE fiber due to homogeneous tension, whereas both the total strain $\varepsilon_{\mathrm{tot}}$ and the light-driven contraction $\varepsilon_{L}$ are not.

For a small deformation of the LCE fiber, it can be assumed that the tension F(t) of the LCE fiber is proportional to the elastic strain $\varepsilon_{e}$,

(6) $$F(t)=kL_{0}\left[\varepsilon_{\mathrm{tot}}(X,t)+\varepsilon_{L}(X,t)\right],$$

where k is the spring constant.

We further assume that the light-driven contraction $\varepsilon_{L}$(*X*,*t*) in the LCE fiber under a small deformation can be expressed as a linear function of the number fraction of *cis*-isomers φ(X,t),

(7) $$\varepsilon_{L}\left(X,t\right)=C_{0}\phi \left(X,t\right)$$

where $C_{0}$ is the contraction coefficient.

To simplify the analysis, we define the total strain $\varepsilon_{\mathrm{tot}}\left(X,t\right)$ as follows: $\varepsilon_{\mathrm{tot}}\left(X,t\right)=\lambda \left(X,t\right)-1=\frac{dx-dX}{dX}$. This is to redefine the deformation gradient λ(X,t), as follows, for simplicity:

(8) $$\lambda (X,t)=\frac{dx(X,t)}{dX}$$

Therefore, F(t) in Equation (6) can be expressed as follows:

(9) $$F\left(t\right)=kL_{0}\left[\lambda \left(X,t\right)-1+C_{0}\phi \left(X,t\right)\right]$$

By integrating both sides of Equation (9) from 0 to $L_{0}$, we can obtain the following expression:

(10) $$F\left(t\right)=k\left[L\left(t\right)-L_{0}+C_{0}\int_{0}^{L_{0}}\phi \left(X,t\right)dX\right]$$

### 2.3. Dynamic LCE Model

The widely accepted dynamic LCE model, originally proposed by Finkelmann et al. [69,70], is employed for calculating the number fraction φ(X,t) of *cis*-isomers in the LCE fiber mentioned in Equation (11). This approach is also adopted by Yu et al., who reported that the induction of *trans*-to-*cis* isomerization in an LCE occurs when exposed to UV or laser radiation with a wavelength shorter than 400 nm. [71]. The number fraction φ(X,t) of *cis*-isomers is determined by the combined effects of thermal excitation from *trans* to *cis*, thermally driven relaxation from *cis* to *trans*, and light-driven relaxation from *trans* to *cis*. It is generally accepted that the thermal excitation from *trans* to *cis* can be disregarded when compared to the light-driven excitation, as indicated by previous research [69]. Thus, the commonly accepted governing equation describing the number fraction is as follows:

(11) $$\frac{\partial \phi (X,t)}{\partial t}=\eta_{0}I(X,t)\left[1-\phi (X,t)\right]-\tau_{0}^{-1}\phi (X,t).$$

In the equation, $\eta_{0}$ represents the light absorption constant, $\tau_{0}$ represents the thermal relaxation time from *cis* to *trans*, and I(X,t) is the current light intensity.

To determine whether the material point *X* is in an illuminated or dark area, it is necessary to compute the current position *x*(*X*,*t*) as specified in Equation (11). By combining Equations (9) and (10), λ(X,t) can be expressed as follows:

(12) $$\lambda \left(X,t\right)=\frac{1}{L_{0}}\left[L\left(t\right)-L_{0}+C_{0}\int_{0}^{L_{0}}\phi \left(X,t\right)dX\right]+1-C_{0}\phi \left(X,t\right)$$

From Equations (8) and (12), we can obtain

(13) $$dx\left(X,t\right)=\left\{\frac{1}{L_{0}}\left[L\left(t\right)-L_{0}+C_{0}\int_{0}^{L_{0}}\phi \left(X,t\right)dX\right]+1-C_{0}\phi \left(X,t\right)\right\}dX$$

By performing integration on both sides of Equation (13) over the range from 0 to *X*, we can derive the following expression:

(14) $$x\left(X,t\right)=\frac{X}{L_{0}}\left[L\left(t\right)-L_{0}+C_{0}\int_{0}^{L_{0}}\phi \left(X,t\right)dX\right]+X-C_{0}\int_{0}^{X}\phi \left(X,t\right)dX$$

The calculated value of *x* from Equation (14) can be used to determine whether the material point of the LCE fiber is in illumination or darkness. Due to the light penetration, the current light intensity *I*(*X*,*t*) of the connecting segment with the roller may be non-uniform for the LCE fibers. For simplicity, we assume that the light penetration depth is much larger than the length of the connecting segment, and in this study, the current light intensity is assumed to be uniform for the connecting segment. Therefore, under illumination conditions, the current light intensity is set as *I*(*X*,*t*) = $I_{0}$, i.e., *x*(*X*,*t*) > *L*(*t*), and *I*(*X*,*t*) = 0 is for the connecting segment in darkness, i.e., *x*(*X*,*t*) < *L*(*t*).

### 2.4. Nondimensionalization

To simplify the calculation, we introduce the following dimensionless quantities: $\bar{t}=t/\tau_{0}$, $\bar{g}=g\tau_{0}^{2}/L_{0}$, $\bar{F}\left(\bar{t}\right)=F\left(t\right)L_{0}\tau_{0}^{2}/J$, $\bar{\beta }=\beta \tau_{0}/J$, $\bar{R}=R/L_{0}$, $\bar{X}=X/L_{0}$, ${\bar{I}}_{0}=I_{0}\eta_{0}\tau_{0}$, $\bar{k}=k\tau_{0}^{2}L_{0}^{2}/J$.

By applying nondimensionalization, we can express Equations (1)–(3), (10), (11), and (14) in the following form:

(15) $$\ddot{\theta }\left(\bar{t}\right)=2/3\bar{g}\sin\alpha /\bar{R}-2/3\bar{F}\left(\bar{t}\right)/\bar{R}-2/3\bar{\beta }\dot{\theta }\left(\bar{t}\right)/\bar{R}$$

For simplicity, $\bar{g}\sin\alpha$ is reduced to ${\bar{g}}_{//}$, where $\bar{F}\left(\bar{t}\right)$ is expressed as

(16) $$\bar{F}\left(\bar{t}\right)=\bar{k}\left[\bar{L}\left(\bar{t}\right)-1+C_{0}\int_{0}^{1}\phi \left(\bar{X},\bar{t}\right)d\bar{X}\right]$$

The length of the current LCE fiber, denoted as $\bar{L}\left(\bar{t}\right)$, can be readily determined using the following straightforward calculation:

(17) $$\bar{L}\left(\bar{t}\right)=1+\theta \left(\bar{t}\right)\bar{R}$$

The value of $\phi \left(\bar{X},\bar{t}\right)$ can be calculated as follows:

(18) $$\frac{\partial \phi \left(\bar{X},\bar{t}\right)}{\partial \bar{t}}=\bar{I}\left(\bar{X},\bar{t}\right)\left[1-\phi \left(\bar{X},\bar{t}\right)\right]-\phi \left(\bar{X},\bar{t}\right)$$

We can rewrite the expression as follows to determine the dimensionless instantaneous position $\bar{x}\left(\bar{X},\bar{t}\right)$

(19) $$\bar{x}\left(\bar{X},\bar{t}\right)=\bar{X}\left[\bar{L}\left(\bar{t}\right)-1+C_{0}\int_{0}^{1}\phi \left(\bar{X},\bar{t}\right)d\bar{X}\right]+\bar{X}-C_{0}\int_{0}^{\bar{X}}\phi \left(\bar{X},\bar{t}\right)d\bar{X}$$

Equations (15) and (18) govern the self-rolling of the roller with an LCE fiber under steady illumination. To determine the current tension ${\bar{F}}_{i}$ of the LCE fiber at a given time, we employ Equations (16) and (17) where the temporal evolution of the number fraction of *cis*-isomers is linked with the angular position displacement of the self-rolling roller. The differential equations with variable coefficients are solved utilizing the Runge–Kutta method, and numerical computations are conducted using Matlab 2016b software. This allows us to obtain the number fraction of *cis*-isomers $\phi_{i}$ and the position of the roller $\theta_{i}$ at the given time $t_{i}$, and subsequently determine the current tension of the LCE fiber. Therefore, the angular displacement of the roller $\theta_{i+1}$ at time ${\bar{t}}_{i+1}$ can be determined by applying Equation (15). Furthermore, the current position ${\bar{x}}_{i}\left(\bar{X},{\bar{t}}_{i}\right)$ of the roller is utilized to estimate the light intensity $\bar{I}\left(\bar{X},{\bar{t}}_{i}\right)$ according to Equation (19). By employing Equation (18), we can subsequently calculate the number fraction $\phi_{i+1}$ of *cis*-isomers present in the LCE fiber. By performing iterative calculations using these equations and a set of given parameters, we can numerically calculate the dynamics of the self-rolling process $\phi_{i+1}$.

## 3. Two Motion Regimes and Mechanism of Self-Rolling

This section focuses on the analysis of the governing Equations (15) and (18), which allow us to identify two distinct motion regimes of the roller: the static regime and the self-rolling regime. We will provide a detailed explanation of the self-rolling mechanism based on the solutions obtained from these equations.

### 3.1. Two Motion Regimes

In order to investigate the motion regimes of the roller under steady illumination of an LCE fiber, it is essential to determine the characteristic values of the dimensionless parameters in the model. We extract the typical material properties and geometric parameters from previous experiments [22,72,73], and compile them in Table 1. The corresponding dimensionless parameters associated with these values can be found in Table 2.

**Figure 3:**
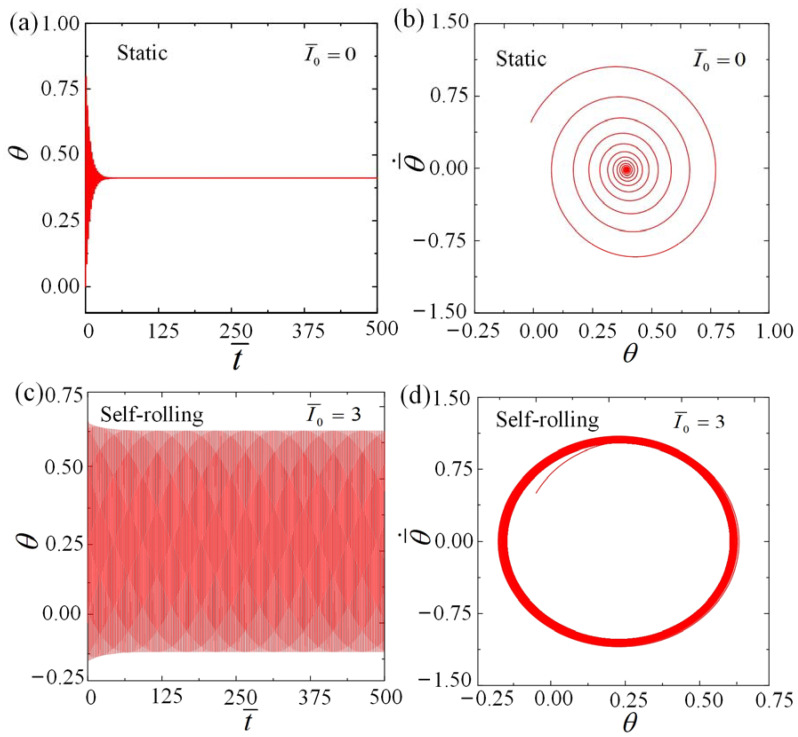
The time evolutions and phase trajectories of the roller are displayed for the two different motion regimes. (**a**,**b**) Static regime with ${\bar{I}}_{0}=0$. (**c**,**d**) Self-rolling regime with ${\bar{I}}_{0}=3$. The other parameters are $C_{0}=0.36$, $\bar{\beta }=0.085$, $\bar{k}=9.8$, $\theta_{0}=0$, ${\dot{\bar{\theta }}}_{0}=0.5$, $\bar{R}=0.2$, and ${\bar{g}}_{//}=0.942$. Under steady illumination, the LCE roller has the potential to transition into two different motion states: the static state and the self-rolling state.

By utilizing Equations (15)–(19), we are able to derive the time histories and phase trajectories for the various motion regimes exhibited by the roller, among which the cases for ${\bar{I}}_{0}=0$ and ${\bar{I}}_{0}=3$ are shown in Figure 3 and Video S1. During the calculation, we assign fixed values to the remaining parameters as follows: $C_{0}=0.36$, $\bar{\beta }=0.085$, $\bar{k}=9.8$, $\theta_{0}=0$, ${\dot{\bar{\theta }}}_{0}=0.5$, and ${\bar{g}}_{//}=0.942$. For ${\bar{I}}_{0}=0$, As depicted in Figure 3a,b, the angular displacement θ amplitude gradually diminishes over time due to damping dissipation. Eventually, the roller settles into a stationary position known as the static regime. For ${\bar{I}}_{0}=3$, as shown in Figure 3c,d, the roller starts moving from rest, and the amplitude of the angular displacement θ gradually increases with time, eventually attaining stability. Finally, when subjected to steady illumination, the roller exhibits continuous and periodic rolling, which is referred to as the self-rolling regime.

### 3.2. Mechanism of Self-Rolling

**Figure 4:**
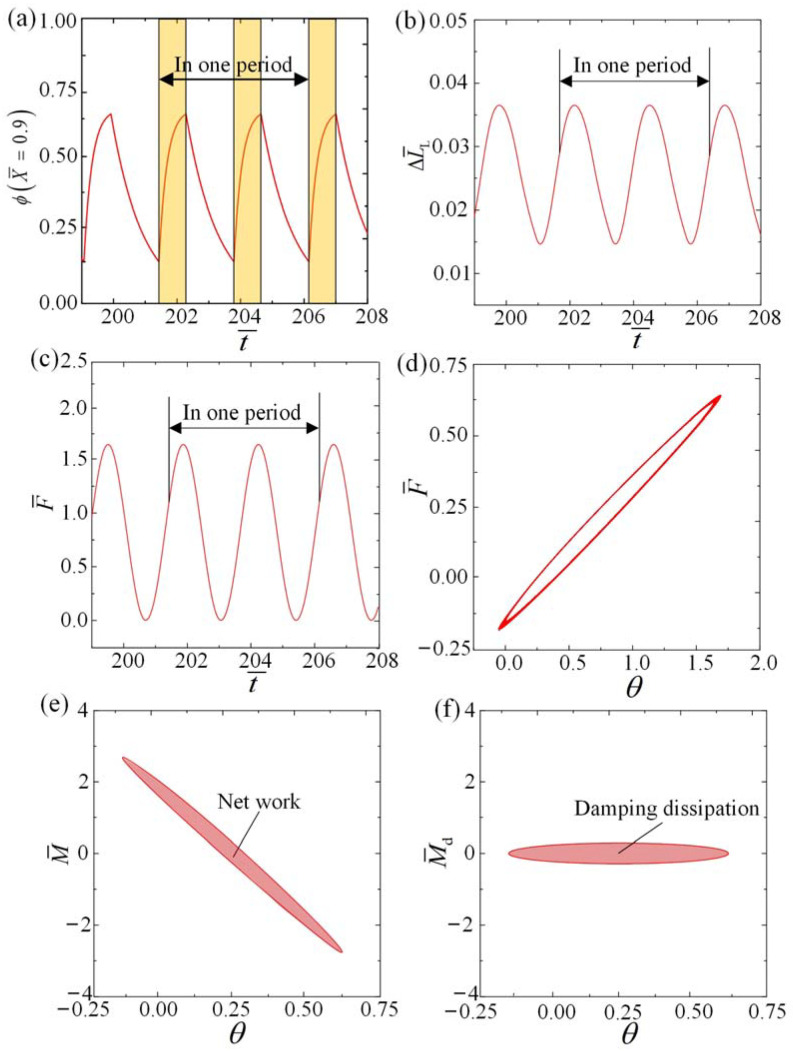
The self-rolling mechanism for the typical cases depicted in Figure 3c,d can be explained as follows: (**a**) The time history curve illustrating the variation in the number fraction of *cis*-isomers at a specific time point. In the figure, the yellow blocks represents that the material point is in the illumination zone. (**b**) The time history curve showing the changes in length of the LCE fiber solely induced by light. (**c**) The time history curve representing the changes in length of the LCE fiber. (**d**) The relationship between tension and angular displacement during one period. (**e**) The relationship between the rolling moment and the angular displacement. (**f**) The relationship between the damping moment and the angular displacement. The net work performed by the elastic force, represented by the region bounded by the curve in Figure 4d, is equivalent to the energy dissipated by the damping. This signifies the sustaining of the self-rolling pattern.

**Figure 5:**
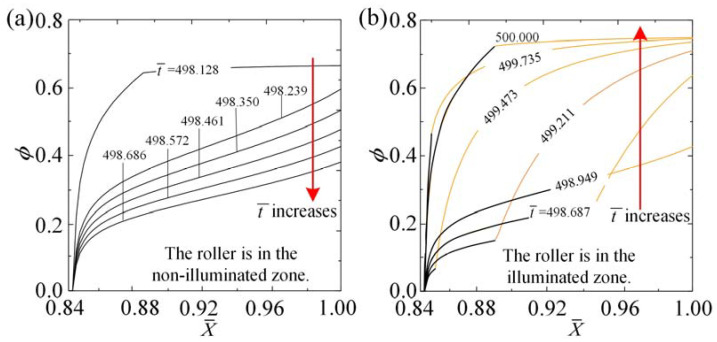
The transformation of the non-uniform number fraction of *cis*-isomers in the LCE fiber, from non-stretched state to stretched state, can be observed in the cases depicted in Figure 3c,d. (**a**) In the non-illuminated zone, the roller’s behavior is depicted. The variation in the number fraction of *cis*-isomers is represented by the black curve. (**b**) In the illuminated zone, the roller’s behavior is illustrated. The part of the fiber in the illuminated zone is shown by the orange curve, while the part in the non-illuminated zone is shown by the black curve.

**Figure 6:**
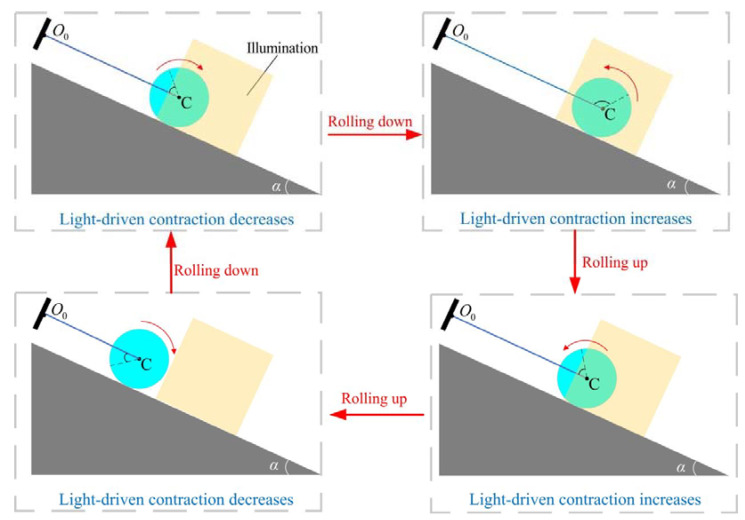
The rolling state of the self-rolling roller in one cycle under the conditions of Figure 3c,d. The dark blue line represents the LCE fiber, the light blue disc represents the roller, the light yellow area represents the illuminated zone, and the dark gray area represents the inclined surface. Under steady illumination, the self-rolling roller will exhibit a continuous periodic rolling.

In order to examine the self-rolling mechanism of the roller, Figure 4 showcases several important physical parameters of the LCE fiber in representative cases depicted in Figure 3c,d. Figure 4a illustrates the temporal evolution of the number fraction of *cis*-isomers ϕ at a specific material point $\bar{X}=0.9$ on the LCE fiber, indicating a periodic variation over time. As the material point enters the illuminated zone, ϕ gradually increases and approaches a maximum value, while ϕ decreases gradually in the non-illuminated zone. Figure 5a,b provides additional visualizations of the non-uniform distribution of *cis-isomer* fractions on the LCE fiber, specifically in the illuminated and non-illuminated regions, respectively, throughout a single rolling cycle. A deeper understanding of this phenomenon can be gained by referring to Figure 6. Figure 4b,c shows the time history curves of fiber length change $\Delta {\bar{L}}_{L}=C_{0}\int_{0}^{1}\phi (\bar{X},\bar{t})d\bar{X}$ and tension in LCE fiber $\bar{F}$ induced purely by light, both of which display periodic behaviors that arise as a result of the periodic variations in the number fraction of *cis*-isomers ϕ.

The relationship between tension $\bar{F}$ and angular displacement θ for the situations shown in Figure 3c,d is further elucidated in Figure 4d. During the process of forward rolling, the tension $\bar{F}$ experiences an increase as a result of the light-driven contraction and the elongation of the LCE fiber length. Conversely, during backward rolling, the tension $\bar{F}$ gradually decreases due to the diminishing light-driven contraction and the reduction in LCE fiber length. The relationship between the rolling moment $\bar{M}$ and the angular displacement θ is further elucidated in Figure 4e, which is determined by both tension $\bar{F}$ and angular displacement θ. The region bounded by the curve in the figure corresponds to the positive net work performed by the LCE fiber, which has been calculated to be 0.3485. In Figure 4f, the relationship between the damping moment ${\bar{M}}_{d}$ and the angular displacement θ is illustrated, with the enclosed area representing the dissipation of damping, also calculated to be 0.3485. Remarkably, the net work carried out by the LCE fiber is exactly equal to the dissipation of damping, suggesting that the self-rolling phenomenon emerges from the interaction between the net work performed by the LCE fiber and the damping dissipation.

## 4. Influence of System Parameters on the Self-Rolling

Within the mechanical model described above for the self-rolling roller, there exist six dimensionless system parameters: $C_{0}$, ${\bar{I}}_{0}$, $\bar{\beta }$, ${\dot{\bar{\theta }}}_{0}$, ${\bar{g}}_{//}$, $\bar{R}$, and $\bar{k}$. In this section, we will conduct a comprehensive analysis of how these system parameters impact the triggering conditions, frequency, and amplitude of the self-rolling phenomenon.

### 4.1. Effect of the Contraction Coefficient

**Figure 7:**
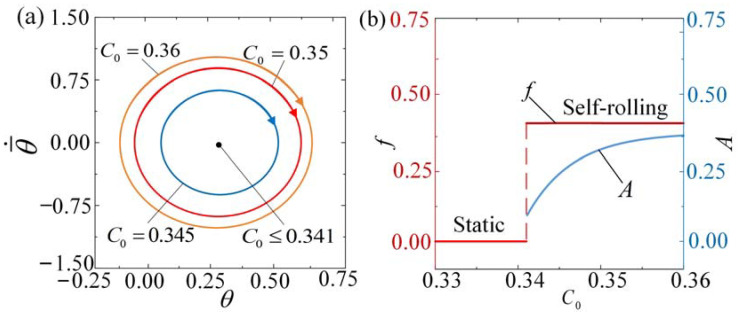
The impact of the contraction coefficient on self-rolling when ${\bar{I}}_{0}=3$, ${\dot{\bar{\theta }}}_{0}=0.5$, $\bar{\beta }=0.085$, $\bar{k}=9.8$, ${\bar{\theta }}_{0}=0$, $\bar{R}=0.2$, and ${\bar{g}}_{//}=0.942$. (**a**) Limit cycles. (**b**) Frequency and amplitude. As $C_{0}$ increases, the frequency of self-rolling remains constant, while the amplitude tends to increase.

Figure 7 and Video S2 show the influence of contraction coefficient $C_{0}$ on the self-rolling roller. In the calculation, we set ${\bar{I}}_{0}=3$, ${\dot{\bar{\theta }}}_{0}=0.5$, $\bar{\beta }=0.085$, $\bar{k}=9.8$, ${\bar{\theta }}_{0}=0$, $\bar{R}=0.2$, and ${\bar{g}}_{//}=0.942$. For $C_{0}$ > 0.341, the limit cycle of light-driven vibration presents a closed loop, indicating the initiation of the self-rolling regime. However, for $C_{0}$ < 0.341, the limit cycles approach the static regime and are represented by the central point in Figure 7a. This outcome suggests that there exists a critical value of $C_{0}\approx$ 0.341 that is necessary to trigger the self-rolling phenomenon. Thorough numerical calculations indicate that the determination of this critical value relies on the specific combination of geometric and material parameters. By taking into account the interaction between various energy components, one can grasp the essence of this phenomenon. When $C_{0}$ is small, the light-driven contraction of the LCE fiber is also small. Consequently, the net work resulting from the coupling between LCE deformation and mass vibration is insufficient to offset the dissipation caused by damping. Figure 7b demonstrates how the contraction coefficient $C_{0}$ affects the frequency and amplitude of self-rolling. As $C_{0}$ increases, the amplitude tends to rise, while the frequency remains relatively constant. These findings imply that the practical application of self-oscillating systems heavily relies on the contraction coefficient of optically responsive materials.

### 4.2. Effect of the Light Intensity

**Figure 8:**
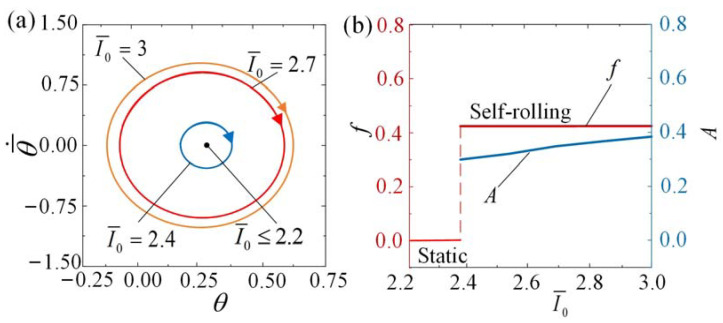
The influence of intensity on self-rolling when $C_{0}=0.36$, ${\dot{\bar{\theta }}}_{0}=0.5$, $\bar{\beta }=0.085$, $\bar{k}=9.8$, ${\bar{\theta }}_{0}=0$, $\bar{R}=0.2$, and ${\bar{g}}_{//}=0.942$. (**a**) Limit cycles. (**b**) Frequency and amplitude. With increasing ${\bar{I}}_{0}$, both the frequency and amplitude of self-rolling present increasing trends.

The influence of light intensity ${\bar{I}}_{0}$ on the self-rolling roller is depicted in Figure 8 and Video S3. In the calculations, the parameters $C_{0}=0.36$, ${\dot{\bar{\theta }}}_{0}=0.5$, $\bar{\beta }=0.085$, $\bar{k}=9.8$, ${\bar{\theta }}_{0}=0$, $\bar{R}=0.2$, and ${\bar{g}}_{//}=0.942$ are set accordingly. Similar to previous findings, detailed numerical calculations indicate the existence of a critical value of ${\bar{I}}_{0}\approx$ 2.2 that triggers the self-rolling phenomenon. This critical value is dependent on the combination of other geometric and material parameters. When the light intensity is set to ${\bar{I}}_{0}\leq$ 2.2, the limit cycles approach the static regime, depicted as a central point in Figure 8a, the self-rolling of the roller can be initiated by a steady illumination for ${\bar{I}}_{0}>$ 2.2. As observed in Figure 8b along with the increases in ${\bar{I}}_{0}$, there are slight increases in self-rolling frequency and amplitude. The phenomenon of self-rolling is a result of the conversion of absorbed light energy into mechanical energy. With increasing light intensity, a larger amount of energy is transformed, resulting in a shorter duration of self-rolling.

### 4.3. Effect of the Damping Coefficient

**Figure 9:**
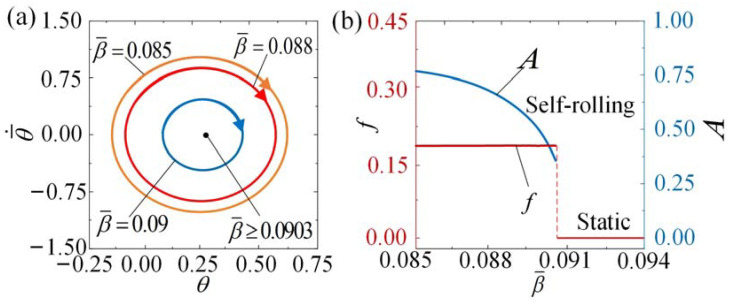
The impact of the damping coefficient on self-rolling when ${\bar{I}}_{0}=3$, ${\dot{\bar{\theta }}}_{0}=0.5$, $C_{0}=0.36$, $\bar{k}=9.8$, ${\bar{\theta }}_{0}=0$, $\bar{R}=0.2$, and ${\bar{g}}_{//}=0.942$. (**a**) Limit cycles. (**b**) Frequency and amplitude. The frequency and amplitude of self-rolling decrease when the damping coefficient $\bar{\beta }$ is increased. However, the decrease in frequency is relatively small.

Figure 9 and Video S4 provide visual representations of how the self-rolling roller is affected by variations in the damping coefficient $\bar{\beta }$. Figure 9a displays the limit cycles corresponding to different damping coefficients, while Figure 9b represents the relationship between the damping coefficient $\bar{\beta }$ and the self-rolling amplitude and frequency. In the calculation, we set ${\bar{I}}_{0}=3$, ${\dot{\bar{\theta }}}_{0}=0.5$, $C_{0}=0.36$, $\bar{k}=9.8$, ${\bar{\theta }}_{0}=0$, ${\bar{g}}_{//}=0.942$, and $\bar{R}=0.2$. A critical value of $\bar{\beta }\approx$ 0.0903 exists. When $\bar{\beta }\geq$ 0.0903, the energy dissipation caused by damping is excessive, and the energy input from the external environment is insufficient to offset this dissipation resulting in a static regime. However, when $\bar{\beta }=$ 0.085, $\bar{\beta }=$ 0.088, and $\bar{\beta }=$ 0.09, self-rolling can be triggered. The self-rolling frequency and amplitude decrease when the damping coefficient is increased, although the decrease in frequency is relatively small. According to the derivation process in this article, with an increase in the damping coefficient, the dissipation of energy becomes more prominent. This leads to a decrease in the amplitude and an extension of the duration of self-rolling within a single period.

### 4.4. Effect of the Spring Constant

**Figure 10:**
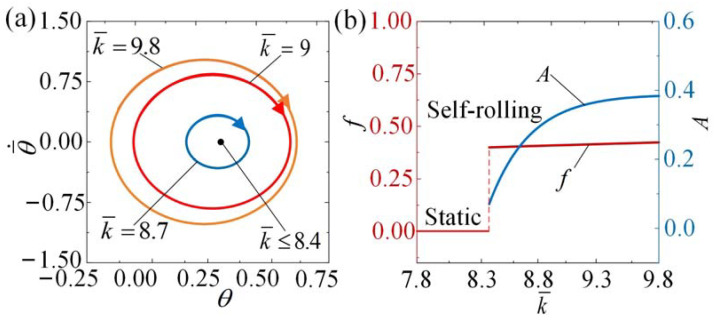
The influence of the spring constant on self-rolling when ${\bar{I}}_{0}=3$, ${\dot{\bar{\theta }}}_{0}=0.5$, $C_{0}=0.36$, $\bar{\beta }=0.085$, ${\bar{\theta }}_{0}=0$, $\bar{g}=1$, $\bar{R}=0.2$. (**a**) Limit cycles. (**b**) Frequency and amplitude. As $\bar{k}$ increases, both the frequency and amplitude of self-rolling exhibit upward trends.

The impact of the spring constant $\bar{k}$ on the self-rolling roller is demonstrated in Figure 10 and Video S5. Figure 10a illustrates the limit cycles corresponding to different spring constants, while Figure 10b displays the variations in self-rolling amplitude and frequency with varying spring constants. In the calculation, we set ${\bar{I}}_{0}=3$, ${\dot{\bar{\theta }}}_{0}=0.5$, $C_{0}=0.36$, $\bar{\beta }=0.085$, ${\bar{\theta }}_{0}=0$, $\bar{R}=0.2$, and ${\bar{g}}_{//}=0.942$. There exists a critical value of $\bar{k}\approx$ 8.4. The roller for $\bar{k}$ ≤ 8.4 is in the static regime, while for $\bar{k}$ > 8.4, the roller behaves in the self-rolling regime. The interplay between different energy terms helps to elucidate this phenomenon. With a decrease in the spring constant $\bar{k}$, there is a corresponding reduction in the tension applied to the LCE fiber, resulting in a decreased amount of work performed by the tension. Therefore, a spring constant greater than the critical value is required for self-rolling to occur. In the static regime, a decrease in the spring constant leads to an increase in the length of the fiber, which subsequently causes a downward shift in the equilibrium position of the roller. Figure 10b demonstrates that the self-rolling amplitude and frequency increase with an increasing spring constant. These findings suggest that a higher spring constant can enhance the dynamic response of self-rolling.

### 4.5. Effect of the Gravitational Acceleration

**Figure 11:**
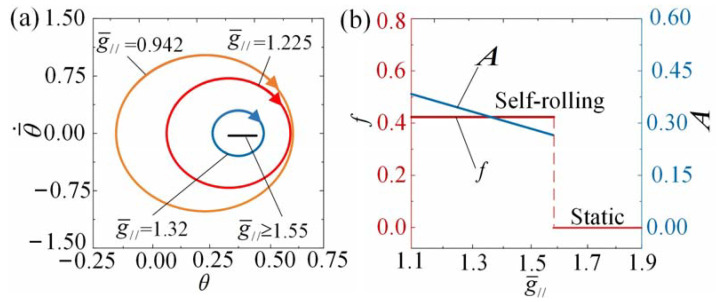
The influence of gravitational acceleration on self-rolling when ${\bar{I}}_{0}=3$, ${\dot{\bar{\theta }}}_{0}=0.5$, $C_{0}=0.36$, $\bar{k}=9.8$, $\bar{\beta }=0.085$, $\bar{R}=0.2$, and ${\bar{\theta }}_{0}=0$. (**a**) Limit cycles. (**b**) Frequency and amplitude. With an increase in ${\bar{g}}_{//}$, the self-rolling frequency demonstrates a minor increment, whereas the amplitude gradually diminishes.

The impact of gravitational acceleration ${\bar{g}}_{//}$ on the self-rolling roller is illustrated in Figure 11 and Video S6. In our calculations, we set ${\bar{I}}_{0}=3$, ${\dot{\bar{\theta }}}_{0}=0.5$, $C_{0}=0.36$, $\bar{k}=9.8$, $\bar{\beta }=0.085$, $\bar{R}=0.2$, and ${\bar{\theta }}_{0}=0$. Figure 11a displays the limit cycles corresponding to different gravitational acceleration, indicating the existence of a critical value of ${\bar{g}}_{//}\approx$ 1.55 to trigger self-rolling. When ${\bar{g}}_{//}\leq$ 1.55, the roller exhibits the self-rolling regime, while when ${\bar{g}}_{//}$ > 1.55, the roller behaves in the static regime. As the gravitational acceleration increases, the length of the fiber correspondingly increases. Consequently, the equilibrium position of the roller shifts lower, causing a larger portion of the fiber to be within the illuminated zone. This, in turn, reduces the portion of the fiber capable of switching between the illuminated and non-illuminated zones, resulting in a decrease in the overall work achieved by the fiber. Notably, in the case of infinite gravitational acceleration where the roller is fully illuminated, the light-driven contraction of the LCE fiber cannot be reversed. As a result, the roller eventually enters the static regime due to the presence of damping. It is clearly seen from Figure 11b that as the dimensionless gravitational acceleration increases, the self-rolling frequency presents a slight increasing trend, while the amplitude exhibits a gradual decreasing trend. The increase in gravitational acceleration has been observed to suppress the dynamic response of the self-rolling mechanism.

### 4.6. Effect of the Radius of the Roller

**Figure 12:**
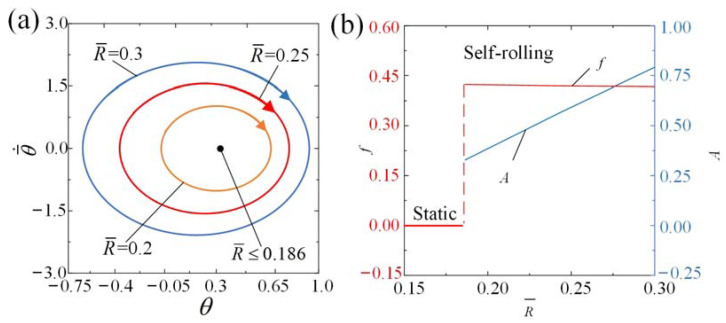
The influence of the radius of the roller on self-rolling when ${\bar{I}}_{0}=3$, ${\dot{\bar{\theta }}}_{0}=0.5$, $C_{0}=0.36$, $\bar{k}=9.8$, ${\bar{g}}_{//}=0.942$, $\bar{\beta }=0.085$, and ${\bar{\theta }}_{0}=0$. (**a**) Limit cycles. (**b**) Frequency and amplitude. The frequency and amplitude of self-rolling increase when the radius of the roller $\bar{R}$ is decreased.

The impact of radius of the roller $\bar{R}$ on the self-rolling roller is illustrated in Figure 12 and Video S7. Figure 12a illustrates limit cycles for different radii of the roller, and Figure 12b represents the functional relationship between the radius $\bar{R}$, amplitude, and frequency. In the calculation, we set ${\bar{I}}_{0}=3$, ${\dot{\bar{\theta }}}_{0}=0.5$, $C_{0}=0.36$, $\bar{k}=9.8$, ${\bar{g}}_{//}=0.942$, $\bar{\beta }=0.085$, and ${\bar{\theta }}_{0}=0$. A critical value of radius $\bar{R}$ approximately equal to 0.186 is present trigger self-rolling. When $\bar{R}\leq$ 0.186, the lower tension of the LCE fiber results in less net work achieved by the rolling moment, and therefore it cannot compensate for the energy dissipated by damping to maintain self-rolling. Figure 12b shows the sightly decreasing trends of frequency and obvious increasing trends of amplitude as the radius $\bar{R}$ increases. This finding suggests that increasing the roller radius can improve self-rolling of the roller under steady illumination.

### 4.7. Effect of the Initial Velocity

**Figure 13:**
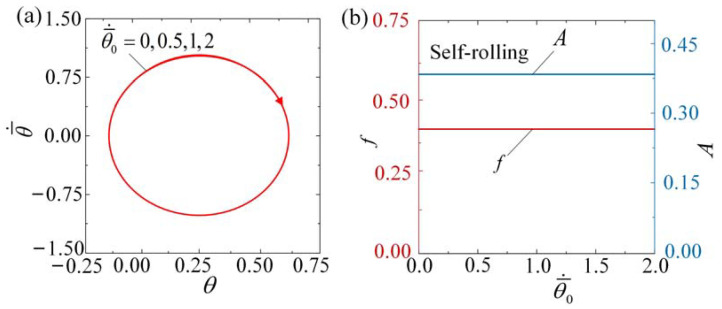
Self-rolling is not affected by the initial velocity when ${\bar{I}}_{0}=3$, $C_{0}=0.36$, $\bar{\beta }=0.085$, $\bar{k}=9.8$, ${\bar{\theta }}_{0}=0$, ${\bar{g}}_{//}=0.942$, and $\bar{R}=0.2$. (**a**) Limit cycles. (**b**) Frequency and amplitude. The self-rolling amplitude and frequency are not influenced by ${\dot{\bar{\theta }}}_{0}$.

The impact of initial velocity ${\dot{\bar{\theta }}}_{0}$ on the self-rolling roller is depicted in Figure 13 and Video S8. In our calculations, we set ${\bar{I}}_{0}=3$, $C_{0}=0.36$, $\bar{\beta }=0.085$, $\bar{k}=9.8$, ${\bar{\theta }}_{0}=0$, ${\bar{g}}_{//}=0.942$, and $\bar{R}=0.2$. It is evident that the initial velocity does not have any effect on the limit cycle, as well as the amplitude and frequency of self-rolling. Self-rolling is triggered at ${\dot{\bar{\theta }}}_{0}=$ 0, 0.5, 1, and 2, with the same limit cycle as shown in Figure 13a. Figure 13b shows that neither the frequency nor the amplitude changes with the variation in ${\dot{\bar{\theta }}}_{0}$. As the transformation from ${\bar{\theta }}_{0}$ to ${\dot{\bar{\theta }}}_{0}$ occurs, it is observed that the conversion of kinetic energy into potential energy remains balanced, and the self-rolling amplitude and frequency, as inherent properties of self-oscillation, remain unaffected by the initial conditions [5].

## 5. Conclusions

Achieving and controlling the desired movements of active machines is generally accomplished through precise control of artificial muscles in a distributed and serialized manner, which is a significant challenge. The emerging motion control strategy based on self-oscillation in active machines has unique advantages including directly harvesting energy from constant ambient light, and no need for complex controllers. Inspired by the traditional tool of the roller, we have developed a new self-rolling system consisting of an LCE fiber and a roller under steady illumination. On this basis, we propose a nonlinear dynamic model for the self-rolling roller under steady illumination and establish the corresponding control equations.

Numerical calculations reveal two motion regimes of the roller, i.e., static self-rolling regimes. Self-rolling of the roller is elucidated to be caused by the light-driven contraction of the LCE fiber under illumination. The self-rolling phenomenon occurs due to the ongoing periodic interaction between light energy and damping dissipation. Additionally, the frequency and amplitude of self-rolling are primarily dependent on various system parameters, such as ${\bar{I}}_{0}$, ${\dot{\bar{\theta }}}_{0}$, $C_{0}$, $\bar{k}$, ${\bar{\theta }}_{0}$, $\bar{\beta }$, $\bar{R}$, and ${\bar{g}}_{//}$. The critical initial velocity ${\dot{\bar{\theta }}}_{0}$ can trigger the self-rolling, but does not affect the self-rolling amplitude and frequency. Increasing the system parameters, including $C_{0}$, ${\bar{I}}_{0}$, $\bar{R}$, and $\bar{k}$, can improve the self-rolling amplitude. In contrast, as ${\bar{g}}_{//}$ and $\bar{\beta }$ increase, the self-rolling amplitude and frequency exhibit a gradual decrease.

In future efforts, the researchers could further develop experimental validation and compare it with predictions. Through experimental verification, the performance and characteristics of the liquid crystal elastomer self-rolling system can be more deeply understood, and the validity of the prediction can be verified. This will help to promote the application and further research of the liquid crystal elastomer self-rolling system. The self-rolling system proposed in this paper exhibits superiorities in terms of simple structure, light weight, speediness, and being an alternative to manual labor. This research endeavor aims to inspire innovative design possibilities for soft robots, energy harvesting devices, micro-mechanisms, and other related fields.

## Figures and Tables

**Table 1:** Material properties and geometric parameters.

Parameter	Definition	Value	Unit
β	Damping coefficient	0~0.001	mg·mm^2^/s
$C_{0}$	Contraction coefficient	0~0.36	/
$\eta_{0}$	Light-absorption constant	0.0003	m^2^/(s·W)
$\tau_{0}$	Thermal relaxation time from *cis*-to-*trans*	0~100	ms
J	Moment of inertia of roller	0~1	mg·mm^2^
$I_{0}$	Light intensity	0~100	kW/m^2^
$g_{//}$	Gravitational acceleration	0~20	m/s
$d_{0}$	Penetration depth	10^−2^	m
$L_{0}$	Original length of LCE fiber	0.1	m
δ	Thickness of LCE fiber	10^−3^	m
E	Elastic modulus	5 × 10^6^	Pa
k	Spring coefficient	1~10	N/m
*R*	Radius of the roller	0~0.3	mm

**Table 2:** Dimensionless parameters.

Parameter	$\theta_{0}$	${\dot{\bar{\theta }}}_{0}$	${\bar{I}}_{0}$	$\bar{\beta }$	$\bar{k}$	${\bar{g}}_{//}$	$C_{0}$	$\bar{R}$
Value	0~50	0~10	0~3	0~0.1	1~10	0~2	0~0.36	0~0.3

## Data Availability

The data that support the findings of this study are available upon reasonable request from the authors.

## Supplementary Materials

The following supporting information can be downloaded at https://www.mdpi.com/article/10.3390/polym15214221/s1, Video S1: Two motion regimes for the roller; Video S2: Effect of the contraction coefficient; Video S3: Effect of the light intensity; Video S4: Effect of the damping coefficient; Video S5: Effect of the spring constant; Video S6: Effect of the gravitational acceleration; Video S7: Effect of the radius of the roller; Video S8: Effect of the initial velocity.
