# Supplementary material for: A Light-Powered Liquid Crystal Elastomer Roller
Source: Polymers (Basel). 2023 Oct 25;15(21):4221. doi: 10.3390/polym15214221 (PMC10650120; doi:10.3390/polym15214221)
Supplement: Supplementary file 1 [file polymers-15-04221-s001.zip › Conflict of Interest.pdf]

Conflict of Interest:

………..

There is no conflict of interest about this paper.

Ethical Statement:

……….

It is conducted according to ethical standards

Acknowledgements:

……………………………………………………………………………………………………

This study is supported by University Natural Science Research Project of Anhui Province (Nos. 2022AH020029 and KJ2020A0453), National Natural Science Foundation of China (Nos. 12172001 and 12202002), and Anhui Provincial Natural Science Foundation (Nos. 2208085Y01 and 2008085QA23).

Funding Body:

………..

The University Natural Science Research Project of Anhui Province
The National Natural Science Foundation of China
The Anhui Provincial Natural Science Foundation
