# Supplementary material for: A Light-Powered Liquid Crystal Elastomer Roller
Source: Polymers (Basel). 2023 Oct 25;15(21):4221. doi: 10.3390/polym15214221 (PMC10650120; doi:10.3390/polym15214221)
Supplement: Supplementary file 1 [file polymers-15-04221-s001.zip › Highlights.pdf]

Highlights

1. A novel light-powered self-rolling liquid crystal elastomer (LCE) roller is established.

2. The rolling of the light-powered LCE roller has two motion regimes: self-rolling regime and static regime.

3. The critical conditions for triggering the self-rolling are numerically obtained.

4. The amplitude of the self-rolling can be controlled by several parameters.
